# Animal reservoirs of SARS-CoV-2: calculable COVID-19 risk for older adults from animal to human transmission

**DOI:** 10.1007/s11357-021-00444-9

**Published:** 2021-08-30

**Authors:** Teresa G. Valencak, Anna Csiszar, Gabor Szalai, Andrej Podlutsky, Stefano Tarantini, Vince Fazekas-Pongor, Magor Papp, Zoltan Ungvari

**Affiliations:** 1 College of Animal Sciences, Zhejiang University, Hangzhou, China; 2 Department of Biosciences, Paris Lodron University Salzburg, Hellbrunnerstrasse 34, 5020 Salzburg, Austria; 3 Vascular Cognitive Impairment and Neurodegeneration Program, Center for Geroscience and Healthy Brain Aging, Department of Biochemistry and Molecular Biology, University of Oklahoma Health Sciences Center, Oklahoma City, OK USA; 4 International Training Program in Geroscience, Doctoral School of Basic and Translational Medicine/Department of Translational Medicine, Semmelweis University, Budapest, Hungary; 5 Department of Biomedical Sciences, Burrell College of Osteopathic Medicine, Las Cruces, NM USA; 6 Institute of Arctic Biology, University of Alaska, Fairbanks, AK USA; 7 International Training Program in Geroscience, Doctoral School of Basic and Translational Medicine/Department of Public Health, Semmelweis University, Budapest, Hungary; 8 Department of Health Promotion Sciences, College of Public Health, University of Oklahoma Health Sciences Center, Oklahoma City, OK USA

**Keywords:** Coronavirus, Immunosenescence, Aging, Zoonosis, Zoo animals, Agricultural animals

## Abstract

The current COVID-19 pandemic, caused by the highly contagious respiratory pathogen SARS-CoV-2 (severe acute respiratory syndrome coronavirus 2), has already claimed close to three million lives. SARS-CoV-2 is a zoonotic disease: it emerged from a bat reservoir and it can infect a number of agricultural and companion animal species. SARS-CoV-2 can cause respiratory and intestinal infections, and potentially systemic multi-organ disease, in both humans and animals. The risk for severe illness and death with COVID-19 significantly increases with age, with older adults at highest risk. To combat the pandemic and protect the most susceptible group of older adults, understanding the human-animal interface and its relevance to disease transmission is vitally important. Currently high infection numbers are being sustained via human-to-human transmission of SARS-CoV-2. Yet, identifying potential animal reservoirs and potential vectors of the disease will contribute to stronger risk assessment strategies. In this review, the current information about SARS-CoV-2 infection in animals and the potential spread of SARS-CoV-2 to humans through contact with domestic animals (including dogs, cats, ferrets, hamsters), agricultural animals (e.g., farmed minks), laboratory animals, wild animals (e.g., deer mice), and zoo animals (felines, non-human primates) are discussed with a special focus on reducing mortality in older adults.

## Introduction

As we are writing this update, it is early April 2021. We have made it through twelve very difficult months after the World Health Organization (WHO) declared the pandemic caused by the novel severe acute respiratory syndrome coronavirus 2 (SARS-CoV-2).

At this moment, the exact origin of the SARS-CoV-2 pandemic that started out from the Wuhan prefecture in China is not fully understood yet. Although it is impossible to exclude the possibility of voluntary manipulation of the SARS-COV-2 virus, the zoonotic transmission seems to be far more likely. [1] Namely, genome sequencing revealed 96% concordance between human the SARS-CoV-2 virus and SARS-CoV-like strains isolated from bats thus strongly confirming that SARS-CoV-2 originates from bats as primary hosts. [1] The spike proteins found on the surface of these bat strains, however, show a weak affinity towards human angiotensin-converting enzyme 2 (ACE) receptors. [1] The zoonotic transmission is still plausible, as other SARS-CoV-like pathogens identified in Malayan pangolins—which were illegally smuggled into Guangdong province—show a much higher affinity to human receptors. [1]

As of April 7, 2021, there were 132,768,361 cases and 2,880,566 victims of the coronavirus disease 2019 (COVID-19) with many more undiagnosed cases worldwide, and there is much fear that we still will have to bemoan many more to come before the longed-for end of the pandemic despite the ongoing vaccination efforts worldwide. [2] According to the Centers for Disease Control and Prevention (CDC), the SARS-CoV-2 virus is primarily a respiratory virus passed on with droplets produced by coughing, sneezing, or speaking in symptomatic patients while there is a considerable percentage of patients where the infection does not cause any of the described symptoms but it is still possible that they transmit the virus. [3] The average incubation period of the disease is estimated to be 5 days and almost all patients exhibit symptoms by day 12. [4] Although most cases manifest themselves as a mild-to-moderate disease, 14% of patients develop a more severe form of the disease, and another 5% fall in the critical category. [5] Estimates show that the case fatality rate of the disease could be as high as 1%, which is almost 10 times higher than that of the seasonal influenza. [5] Aging seems to play crucial role affecting disease severity and mortality, [6] followed by other factors, such as concurrent number of comorbidities and lifestyle factors, for instance smoking. [5]

The array of symptoms differs between hospitalized and non-hospitalized patients. Among hospitalized patients, the most frequent symptoms appear to be fever (90% of patients), cough (75%), and dyspnea (50%). [7] In contrast, non-hospitalized patients exhibit cough (12%) most frequently, followed by fever (10%), then myalgia (8%), and fatigue (6%). [7] Other symptoms, such as loss of smell and taste, appeared in 1% of the infected adult population, whereas psychiatric illnesses, like anxiety and depression, affected approximately 16% and 4% of patients, respectively. [7] More severe manifestations of the disease include pneumonia, acute respiratory distress syndrome, acute liver injury, cardiac injury, acute cerebrovascular disease, and shock. [8]

Studies indicate that certain symptoms and lesions may persist months after the acute COVID-19 infection and are frequently referred to as post-COVID syndrome or “long COVID-19.” Approximately, 40–90% of patients affected by COVID-19 report symptoms, such as fatigue, dyspnea, or neurological complaints weeks after remission. [9] More severe manifestations of the post-COVID syndrome include stroke, microhemorrhages, renal failure, myocarditis, and pulmonary fibrosis. [10] The development of the post-COVID syndrome may be related to the aftereffects of the infection itself, such as residual inflammation and organ damage, to the treatment of the acute infection, such as ventilation, to the social consequences of isolation, or to the effect of the infection on certain pre-existing conditions. [9] Factors, such as extensive lung involvement or number of comorbidities, may be used as predictors for the development of post-COVID syndrome, suggesting that the severity of the acute infection and health status of the patient are likely directly associated to the long-lasting consequences of the disease. [9, 10] The exact duration, manifestations, and characteristics of the post-COVID syndrome are not fully known yet. However, experts suggest that any symptom residing after 3 weeks after remission should be considered as post-COVID syndrome. [11].

## Increased COVID-19 mortality in older adults

**Fig. 1:**
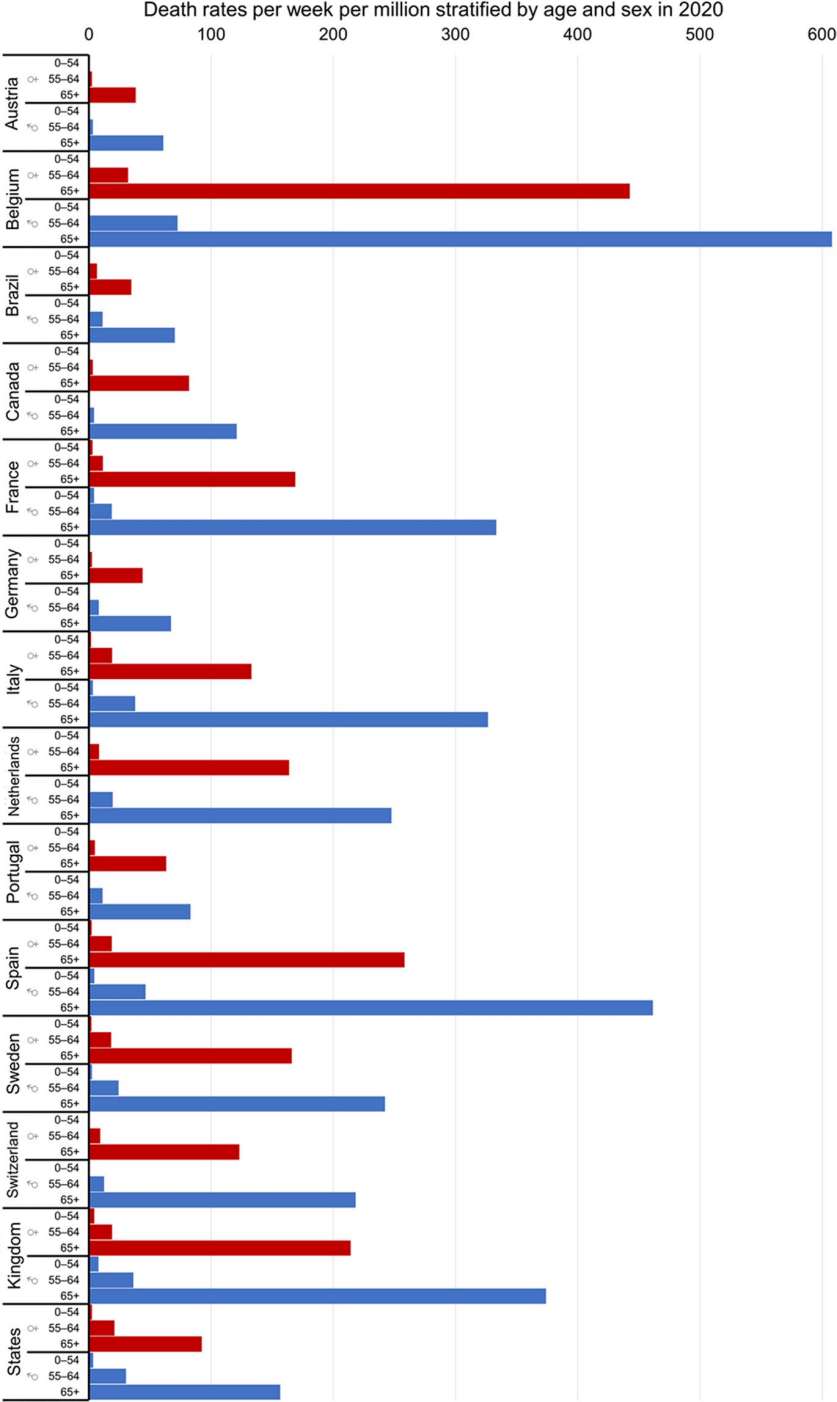
COVID-19 death rates per week per million inhabitants stratified by age and sex for the year 2020. Data are re-plotted from reference [22]

Mortality rates of COVID-19 gradually increase with age and exhibit a marked surge after age 65 (Fig. 1). [6, 12–24] There are also sex differences in regards of the magnitude of mortality rates with men exhibiting worse results compared to female patients. [25] Mortality rates are also influenced by geographical location as seen in Fig. 1. [25, 26] Studies indicate that the age distribution of a population may explain up to two thirds of the variance of mortality rates observed between different countries. [15] In Europe, the higher mortality rates may be attributed to the higher occurrence of outbreaks in nursing homes. [26].

The causes of these age-related differences in mortality rates are not yet fully understood. [6] Possible causes likely include the less efficient functioning and coordination of both cellular and molecular elements of the immune system, the higher number of comorbidities, and the overall frailty and impaired organismal and cellular resilience of elderly patients. [6, 27–30].

Since it got obvious at the beginning of the pandemic that older adults would be the susceptible age group when contracting COVID-19, public health authorities and governments on the whole globe aimed at limiting the transmission risk for the elderly. Thus, people were prompted to shut down and avoid social activities and gatherings, populated areas, and means of public transportation. Likewise, nursing homes and assisted living facilities for seniors were locked down from outside visitors including family members. While these drastic measures were implemented for relatively short time periods in spring 2020, when all social activities including church services were canceled, the second and the third so-called lockdowns of most European countries including Germany, France, and the UK were implemented for several weeks. All these steps were and are effective and limit spread of COVID-19 by reducing the number of infections. [31, 32].

## Zoonotic spread of SARS-CoV-2 causing COVID-19

It was clear from the beginning that COVID-19 is a zoonotic disease and that coronaviruses broadly use bats as their primary hosts without causing heavy symptoms of disease in them. [33] However, in between the primary hosts, most likely horseshoe bats from the genus *Rhinolophus* spp. and the human host, [33] coronaviruses generally use another species as intermediate host before spreading on to humans. [33] Contrary to the bats, the intermediate hosts often show symptoms of weakness and disease and thus may similarly be affected as humans. Even after 1 year of intense worldwide research, it remains to be shown where exactly and when SARS-CoV-2 spread on to humans. Pangolins and snakes are discussed but the picture is far from being clear. [34, 35] Most likely to date, SARS-CoV-2 is a recombinant virus between a bat and a species that has yet to be determined. [35] Many of the early cases were linked to the Huanan seafood market in Wuhan, Hubei province, as we detailed recently. [36] So far, of the 585 environmental samples directly taken from the market area, 33 were reported to be positive for the SARS-CoV-2 by the Chinese Center for Disease Control and Prevention [37] and all were from the market’s western part which is where alive wildlife were sold. [37] However, we are still left in the dark which animals are involved as the origin of the actual pandemic and currently, a WHO research team is seeking that answer directly on the ground in Wuhan.

In the SARS outbreak of 2002–2003, caused by SARS-CoV-1, similar food markets selling alive animals for food consumption were implicated and later, palm civet cats (*Paradoxurus hermaphroditus*), native to South Asia, were shown as intermediate hosts in between horseshoe bats and humans. [38, 39].

For the current SARS-CoV-2 pandemic, Malayan pangolins (*Manis javanica*) seized in anti-smuggling operations in southern China were found to carry very similar coronaviruses and therefore possibly are responsible for the infection of humans in Wuhan last year [40] but no information about symptoms in the animals is available to date. However, coronaviruses are present in many wild mammals in Southern Asia and it remains to be shown which species indeed served as shuttle for transmission ahead the COVID-19 pandemic. In vivo studies suggest that several species, including cats, can be infected with SARS-CoV-2 virus, whereas chickens, pigs, and ducks are not susceptible. [41].

Even more timely in the current situation of the still ongoing pandemic is understanding the immediate transmission risks for SARS-CoV-2. Undoubtedly, the current pandemic is being sustained via human-to-human transmission of SARS-CoV-2. However, due to the zoonotic origin of COVID-19, understanding the animal reservoirs and potential vectors is of great importance for coherent risk assessment strategies. Thus, we are updating the information available on animal-to-human transmission and human-to-animal transmission for SARS-CoV-2 with a special focus on the elderly and their specific situation.

### Companion animals as reservoirs of SARS-CoV-2

**Fig. 2:**
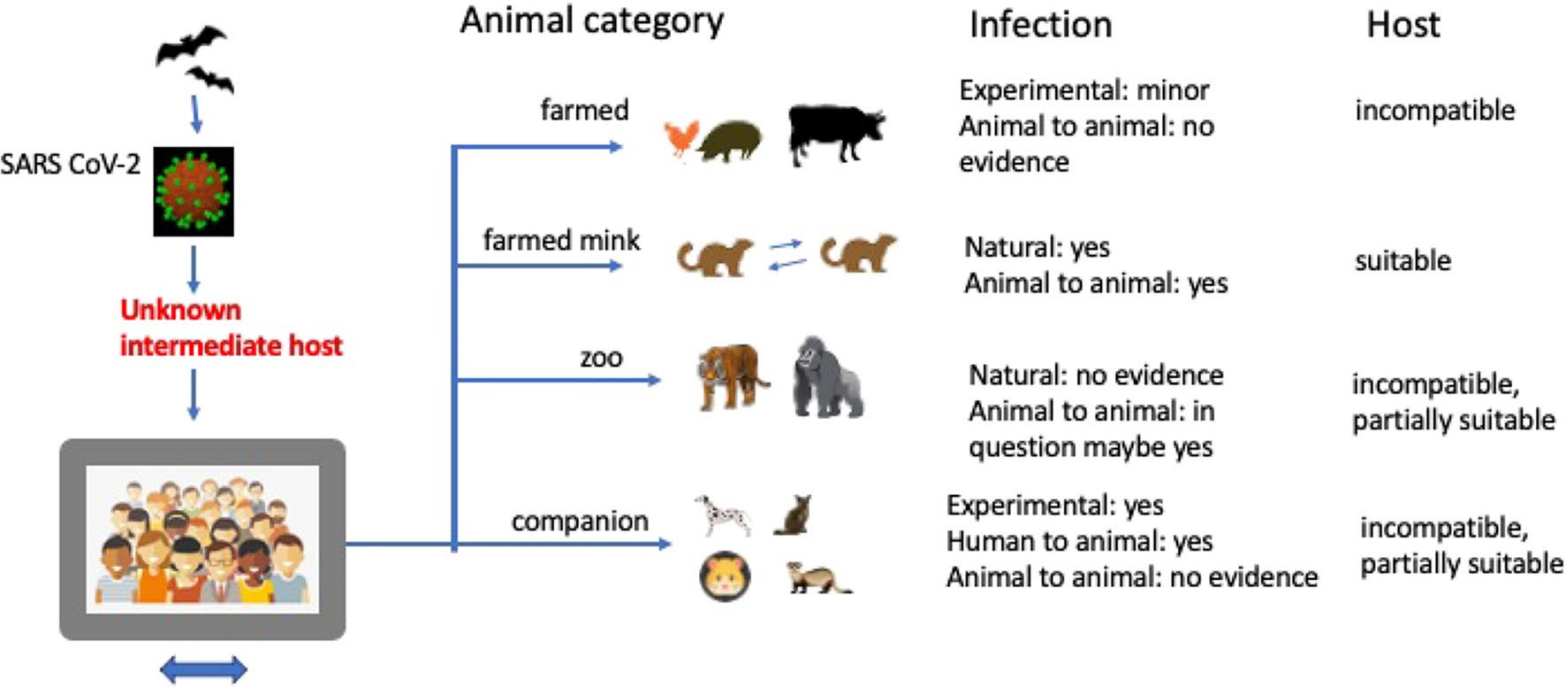
Role of farmed, zoo, and companion animals as putative hosts for infection or routes of transmission with SARS-CoV-1 and SARS-CoV-2. Notably, mustelids and farmed mink are special examples

Susceptibility of cats [32, 39–52] and dogs [53–56] to SARS-CoV-2 infection is supported by several observations. Infected human pet owners can passively transmit SARS-CoV-2 via surfaces, the skin, or fur of an animal. In our recent contribution, [36] we summarized the transmission risks from pet dogs and cats to their owners by focusing on the risk for retired, elderly people. Here, we provide an update on the available evidence about SARS-CoV-2 infection in pets (Fig. 2). As with other coronaviruses and SARS-CoV-1 circa 18 years ago, only certain non-human, mammalian hosts can contract the virus and transmit it to humans. Transmission generally is possible in an experimental or in a natural situation. While the experimental infections, carried out in isolated laboratories, are representing no immediate risk for transmission, the natural situation of people sharing their house with pet animals does require scientific attention. Particularly following the early reports from China when there were mixed messages over the anthroponotic risk and people reacted by reportedly abandoning or even killing their pets for fear of an infection. [57] However, the human risk for contracting COVID-19 from their own dogs and cats is minor as we concluded in Csiszar et al. [36] and as summarized in Leroy et al. [58]. In a large study on cats and dogs from Italy, [59] a total of 603 dogs and 316 cats were sampled for a possible COVID-19 infection and showed that 3% of the dogs and 6% of the cats had SARS-CoV-2-neutralizing antibodies with 13% of dogs and 5% of cats from households with known COVID-19 cases. [59] None of the pets showed symptoms of respiratory disease at the time of sampling. [59] Concluding, the above-described detections of the SARS-CoV-2-neutralizing antibodies on pets from already infected households suggest that COVID-19 infections in dogs and cats do occur frequently and that it is likely that infected humans transmit the virus to their pets [58] rather than the other way around. As we will get used to the SARS-CoV-2 virus in the human population sharing their homes with pet companions, further research testing for the virus present in the pets and fully defining their infection status will be useful. It is estimated that there are currently 135 to 184 million pet dogs and cats in the USA (according to the *U.S. Pet Ownership & Demographics Sourcebook* by the American Veterinary Medical Association (AVMA) and the biennial APPA National Pet Owners Survey by the American Pet Products Association, respectively). Thus, given the prevalence of COVID-19 in the USA, it can be expected that thousands to hundreds of thousands of companion animals have been already exposed or potentially even infected. A recent study of stray cats in the city of Zaragoza, Spain, demonstrated that 3.5% of the animals tested positive for SARS-CoV-2, suggesting that stray animals might be especially susceptible to SARS-CoV-2 infection due to pre-existing, concomitant bacterial and virus infections. [55] Thus, people who are suspected or confirmed to be infected with SARS-CoV-2 should therefore minimize close direct contact with animals including companion animals; farm, zoo, or other captive animals; stray animals; and wildlife in order to limit any potential human-animal zoonotic transmission.

In the case of the dogs and domestic cats that have worldwide tested positive for SARS-CoV-2 by having viral RNA in saliva and nose secretions, the picture emerges that while SARS-CoV-2 has the ability to infect different pet species, the viral shedding from pets may usually not be sufficient to in turn infect other family members or other pet animals encountered during walks (summarized in Wang et al. [60]).

As broadly accepted, pets represent an important source for affection, conversation, and activity for seniors. [61] Reportedly, levels of oxytocin increase and anxiety and cortisol levels are calmed in people having a pet. [62] While these findings hold true for the entire population, pet ownership probably was and presently is particularly beneficial for older people during the social isolation due to COVID-19. [63] Reportedly, older people were suffering from symptoms of anxiety, depression, poor sleep quality, and physical inactivity. [63] Amid the COVID-19 pandemic, many people decided to adopt a dog to ease loneliness, improve mood, and receive companionship. [64, 65] Interestingly, as social restrictions became stricter, the dog adoption rate increased in Israel and similar findings were reported from the USA and Europe. [64] Although the greatest risk of COVID-19 exposure remains person to person contact, it should be noted that there is a potential risk for contracting SARS-CoV-2 on the premises of animal shelters due to the above-mentioned possibility that the pet gets infected from its owner and carries the virus on its fur etc. When considering the stability of SARS-CoV-2 on environmental surfaces and excreta, the picture becomes clear that the virus can be very stable in a wide range of pH values at room temperature but it is also susceptible to standard disinfection methods. [66, 67] If a person in the same household becomes sick, it is important that the person is isolated from the others and the pets. If hospitalization of the infected person is required, the pet should preferably be taken care of at home instead of being taken to an animal shelter where other pets from infected households may be taken to. Consequently, susceptible seniors should physically avoid animal adoption centers for the time being to prevent infection. Veterinary practitioners worldwide are warning that the demand for animals during lockdown periods will have major implications for animal welfare because pets from unscrupulous dealers and carrying diseases and pathogen are brought into the system. As much as dogs and cats are a valuable source of comfort for many people throughout the COVID-19 crisis and for millions of people they are an integral part of the family, attention should be given to a pet’s health and hygiene.

The Centers for Disease Control and Prevention (CDC) recently issued an guidance for public health professionals managing the home care and isolation of people with COVID-19 who have pets or other animals (including service or working animals) in the same home and managing companion animals infected with SARS-CoV-2. [68] These guidelines emphasize the close coordination between state and local health officials and the veterinary community if a companion animal is suspected or tests positive for SARS-CoV-2. [68] The guideline states that animals that do not require veterinary treatment or care should be isolated and monitored by their caretakers at home. [68] Due to concerns of potential human-to-animal transmission of SARS-CoV-2, the CDC recommends that people with COVID-19 and in-home isolation should isolate themselves from household animals. In addition, increases hygiene measures and hand washing after close contact with the pet should be implemented. Although it is not legally required for cats and dogs that both the state public health veterinarian and/or state animal health official should be informed of animals that are being tested for SARS-CoV-2, it would be of great use to the veterinary services, especially when animals indeed would be diagnosed with a SARS-CoV-2 infection. [68].

According to these CDC guidelines, confirmatory testing through National Veterinary Services Laboratories (NVSL) is required for all animals except domestic cats and dogs from state, territorial, local, and tribal jurisdictions that have previously confirmed SARS-CoV-2 in cats and dogs. [68] SARS-CoV-2 is a disease reportable to the World Organization for Animal Health (OIE) and the United States Department of Agriculture (USDA) is responsible for reporting positive SARS-CoV-2 cases in animals in the USA to the OIE. [68].

Summarizing, all these studies are positive news for the susceptible group of the seniors who, when infected with SARS-CoV-2, have an increased risk to develop serious pathologies and courses of the disease. It is now clear that many people infected with SARS-CoV-2 remain asymptomatic while spreading the disease. If the same would hold true for companion animals, it could not be ruled out that asymptomatic household pets also could shed the virus and infect people without exhibiting actual symptoms themselves. [36] Yet, pets get infected from their human owners and not vice versa and not a single outbreak cluster arose from an infection with a potentially asymptomatic companion animal [36] as outlined earlier in “Companion animals as reservoirs of SARS-CoV-2” section. The predicted low viral loads detected in dogs and cats suggest that elderly people likely bear no large risk to contract COVID-19 when walking their dogs in nearby parks. We however would like to point out that practicing good hygiene and hand washing post potential exposures and avoiding too close contact with pets further safeguards the vulnerable age group of the seniors.

### Agricultural and carnivoran animals as reservoirs of SARS-CoV-2

Handling farm animals, which are susceptible to SARS-CoV-2 infection, can carry additional risks when large numbers of animals are kept in close contact. Yet, experimental infection studies show that the economically important livestock species such as pigs, poultry, and cattle are not susceptible to infection. [69, 70] It thus seems unlikely that elderly citizens and even those in close contact with agricultural animals could get infected with SARS-CoV-2 due to their contact with the animals. [69] Certainly however, we are only at the beginning of understanding if and how different animals could be affected by SARS-CoV-2. For the vulnerable age group of the seniors, it means that there is no big transmission risk between the most important livestock species and humans. Similar to pigs, poultry, and cattle, also experimentally infected rabbits bear no transmission risk. [43].

Although our common livestock species are not susceptible to infection, there were serious consequences of COVID-19 on the food supply chain. However, these effects were unrelated to a potential infection risk from the animals but rather included restrictions of demand, closing of food facilities, and financial restrictions. [71].

A totally different picture emerges from farmed carnivorous American minks (*Neovison vison*) and ferrets (*Mustela putorius*). [72] Experimental infection induced substantial viral loads, symptoms of rhinitis, and also SARS-CoV-2 reactive antibodies. [69, 72, 73] Transmission therefore may take place between infected humans and ferrets when they are kept as pets and developing symptoms just as recently shown from a pet ferret from Slovenia that got infected from its human owner. [74] In Denmark and in the Netherlands, SARS-CoV-2 circulated between farmed mink and workers on affected mink farms causing similar infection and transmission rates in people as the human variants. [74–76] When switching species, however, SARS-CoV-2 may develop mutations due to host adaptation and in the case of the high population densities in the farmed mink, there quickly was the “cluster 5 variant” identified from Denmark that reportedly was more difficultly eliminated by human antibodies against wild-type SARS-CoV-2. [74] Notably, that variant was detected in a small number of people in one location only, it was not found to spread and it has not been detected since September 2020. Finally, there is no case of infected seniors reported getting exposed to it or even contracting that virus variant. While mink farming was banned or got rare in several European countries already several decades ago, there are more than 200 mink farms in the USA and there were mink deaths reported from farmers in Utah. [72] COVID-19 thus not only greatly affects the human population but also weasel-like carnivorans which are raised for their fur or kept as household pets. Considering animals kept in captivity for fur production, the raccoon dog (*Nyctereutes procyonoides*) may play a potential role as intermediate host since it is very important for the Chinese fur market and was found to be susceptible to an experimental SARS-CoV-2 infection and it was observed that it can infect other animals in close vicinity. [77].

Although much less commonly kept as pets than dogs and cats, ferrets are sociable and affectionate, quiet, and very playful pets. Yet, in the current COVID-19 situation, they seem to be more susceptible for infection although more experimental infection studies are required to undermine the data. For the vulnerable age group of the seniors, the findings of clinical signs and possible transmission in ferrets ask for broad training and cautious measures if there are pet ferrets in the same household and if seniors regularly encounter farmed, wild, or captive mustelids.

### Laboratory animals as reservoirs of SARS-CoV-2, experimental infection studies, and suitable model systems to study vaccine efficacy

With the rapid aging of NIH R01 funded investigators in the USA, there is an increasing number of retirement age scientists working in academia, [78–82] who can be potentially exposed to a wide range of laboratory animal species. [79] Here, we consider the susceptibility of laboratory animals to SARS-CoV-2 infection and possible routes for animal-to-human transmission.

It should be noted that dogs, which are susceptible to SARS-CoV-2 infection (see above), are frequently used in veterinary, geroscience, and cardiovascular research as large animal models of aging, heart failure, and other chronic diseases. [83–89] Not all of these studies are performed in laboratory dogs but rather they are done on privately owned companion dogs whose owners choose to participate in these studies and who should receive special training on potential transmission risks and hygiene measures.

Many scientists who are in contact with dogs on a daily basis in an academic environment and the pharmaceutical industry belong to vulnerable age groups. In the laboratory setting, there is an increased probability for contact with bodily fluids of potentially virus carrier animals, especially during procedures that involve invasive surgery. [90] Research teams should also receive adequate safety training to prevent transmission of SARS-CoV-2 and closely follow laboratory guidelines for handling biological specimens, waste, and hazardous materials.

Researchers working on animal models of COVID-19 infection are especially at risk. Experimental infection studies showed that both golden hamsters (*Mesocricetus auratus*) and Chinese hamsters (*Cricetulus griseus*) are susceptible to SARS-CoV-2 infection, develop similar clinical symptoms to humans and immunity against reinfection, yet not shedding enough viral RNA to be a potential source of zoonotic transmission (Fig. 2). [34, 91].

Despite being suggested as models as to evaluate vaccine efficacy and antiviral therapy, wild-type *Mus musculus* models initially were found to be insignificant systems due to inefficient rates of SARS-CoV-2 virus replication. [92] SARS-CoV-2 has to bind host cells through the angiotensin-converting enzyme 2 (ACE2) protein receptor which seems to differ greatly between humans and mice, and thus, transgenic mice expressing hACE2 were produced to overcome this issue and to enable successful virus replication in experimental animals. [93].

As it became clear that the ACE2 molecule is used by SARS-CoV-2 for cellular entry, computer analysis of predicted ACE2 amino acid sequences of vertebrates was compared to that of humans. [94] Species of the genus *Peromyscus* fall under the Cricetidae family and represent the most common mammals of North America. Research on this genus has been widespread across so many disciplines that the genus has aptly been referred to as “The Drosophila of North American Mammalogy”. [95] Furthermore, peromyscines were identified as hosts for zoonotic diseases serving as reservoirs of the Sin Nombre strain of Hantavirus, [96] *Babesia microti*, and *Borrelia burgdorferi* and rickettsias of *Ehrlichia chaffeensis* and *E. ewingii*. [97, 98] The white-footed mouse, *Peromyscus leucopus*, is no stranger to scientists studying aging in a laboratory environment, as these animals can live up to 8 years in captivity. [99–103] Based on the above unique features of peromyscine rodents, their possible role in reverse zoonosis of SARS-CoV-2 has been the focus of research. [104, 105] Both of these reports showed that *Peromyscus maniculatus*, commonly known as deer mice, are susceptible to infection after an intranasal exposure to a human SARS-CoV-2 isolate, yet do not show signs of clinical distress. Fagre et al. contribute this to the lack of IFN-y or IL6 elevation in contrast to levels detected in fatal COVID-19 cases in humans. [104] Both Fagre et al. and Griffin et al. further demonstrated that the virus can be transmitted by direct contact. [104, 105] Fagre et al. showed that on days 3 and 6 post viral challenge the lungs of the infected animals showed pathological alterations that were resolved by day 14, although low levels of viral RNA was still detected. [104] At the same time, they observed neutralizing antibodies to multiple viral antigens. Fagre et al. also detected virus entry to the brain. [104] Griffin et al. showed that infected deer mice can shed the virus, had low lymphocyte counts and elevated neutrophil levels, following the trends observed in COVID-19 patients. [105] It is important to point out that *Peromyscus* species have been shown to resemble human hematological profiles much better as compared to *Mus musculus* C57BL/6 animals. [106] Although the later species can be humanized at the ACE2 locus, they will still be inferior model based on their hematological profiles when compared to *Peromyscus* species. Both Fagre et al. and Griffin et al. point out that the potential of deer mice undergoing reverse zoonosis of SARS-CoV-2 in the wild is unknown. [104, 105] *Peromyscus* animals are in the juxtaposition of wildlife and laboratory science, since they are not only abundant in nature but also represented as laboratory reared, wild-derived, outbred stocks in several laboratories and in the Peromyscus Genetic Stock Center (https://www.pgsc.cas.sc.edu/).

Interestingly, experimental infection of white-tailed deer (*Odocoileus virginianus*) leads to successful RNA replication of the SARS-CoV-2 virus, due to the shared similarity of the ACE2 protein with humans. [107].

Similarly, non-human primates from the genus *Macaca* and *Callithrix* were experimentally infected with SARS-CoV-2 and were found to have several advantages including the homologous ACE2 receptor protein and a similar virus shedding pattern from the nose and throat to that of humans. [34].

Altogether, over the past year, our knowledge on SARS-CoV-2 has substantially increased with the help of traditional epidemiology and modern biomedicine. The speed at which the new recombinant vaccines against SARS-CoV-2 were produced is impressive and the recent successes may pave the way for many future RNA-based vaccines. Opposite to conventional vaccines where inactivated viral proteins commonly are injected to elicit an immunization process, the new recombinant vaccines contain the mRNA of the viral protein and induce a cascade leading to the synthesis of effective, neutralizing antibodies. The new vaccines may be particularly beneficial for seniors as they seem to have good translation efficacy even in view of the many emerging SARS-CoV-2 mutants.

### COVID-19 in zoo animals

Several cases of COVID-19 infection in zoo animals have been reported during the past year. At the Bronx Zoo in New York City, a Malayan tiger was tested positive for the SARS-CoV-2. [108, 109] In addition, six other big cats (another Malayan tiger, two Amur tigers, and three African lions) were reported to exhibit symptoms, including dry coughs, which are indicative of an infection with SARS-CoV-2. [109] These felines likely have contracted the virus from a caretaker, who was asymptomatic at the time of contact with the animals. [109].

On January 11, 2021, the first transmission of SARS-CoV-2 from humans to great apes such as the gorillas at the San Diego Zoo safari park was reported [110] (https://zoo.sandiegozoo.org). Besides some coughing, the gorillas were doing well and it was suspected that the animals acquired the COVID-19 infection from an asymptomatic staff member despite all security measures. However on January 25th, it was publicized that a 48-year-old gorilla called “Winston,” identified as SARS-CoV-2 positive after examination of fecal samples, had to be treated with heart medication, antibiotics, and monoclonal antibody therapy. [111] COVID-19 monoclonal antibodies have been approved for emergency use and have helped many patients overcome the virus including the gorilla “Winston.” The fact that Winston and some others in the troop at San Diego Zoo safari park got infected is especially alarming because gorillas are under the threat of viral (Ebola) and ecological extinction. [112].

SARS-CoV-2 had been confirmed in nondomestic felids such as tigers (*Panthera tigris jacksoni*, *Panthera tigris altaica*) and lions (*Panthera leo*) first at the Wildlife Conservation Society’s Bronx Zoo in New York City. [113] There also have been reports on COVID-19 in a snow leopard (*Panthera uncia*) in the Louisville Zoo [114] and lions in the Barcelona Zoo [115] and a tiger in a zoo in Sweden, which had to be put down. [116] The virus variants detected in the animal samples were identical to the ones detected in humans suggesting that the animals got infected from staff members. In July 2020, a COVID-19 infection was reported in a zoo puma (*Puma concolor*) in South Africa. [117] All the cases had contact with SARS-CoV-2-positive humans.

The mink family (Mustelidae) was observed to be very susceptible to a COVID-19 infection. [118] Not surprisingly, COVID-19 infections were meanwhile reported from nine European countries and in the USA both in the European mink (*Mustela lutreola*) and the American mink (*Neovison vison*). [117].

The Middle East respiratory syndrome (MERS) is caused by a novel coronavirus that is at least 10 times more deadly than COVID-19. According to Hemida et al. [119], MERS-CoV has been positively identified in dromedary camels since 2010, long before the first human case was reported. Camel-to-human transmission occurred in 2012 in Saudi Arabia in camel herders, including a person who applied a topical medicine to a sick camel. [120, 121] Although millions of camels are kept in captivity in close contact to humans (Kenya alone is home to 3 million camels, which represents nearly 10% of all the camels in the world), no cases of dromedary camels carrying SARS-CoV-2 have been reported yet.

Taken together, we should not underestimate the risk of spreading the disease into “unusual” pools, such as zoo animals. Since in the past few decades, petting zoos are becoming more popular at almost every zoo and many state fairs and exhibitions, special care should be taken to protect groups that are at risk. If elderly citizens and individuals with compromised immune systems, such as cancer patients and organ transplant recipients, are appropriately warned and protected, infections can be prevented. [122] Proactive measures should be developed—such as regular testing of zoo animals, especially in petting zoo sections, and the availability of hand sanitizers. It is also advisable to post special signs to alarm visitors of the risk of COVID-19 infection.

## Detection of SARS-CoV-2 in animals

A year after the outbreak of the pandemic, the detection of SARS-CoV-2 in different types of clinical specimens is well-established. [60] For current guidelines for veterinarians, please visit the website of the American Veterinary Medical Association. [43] Extensive epidemiological surveys of SARS-CoV-2, similar to the Italian study in Lombardy, [59] will be required to monitor seropositivity in companion animals to understand their role in community disease dynamics.

## Conclusions and perspectives

A year after the outbreak of the pandemic, we can conclude that COVID-19 raged through homes for the elderly and took the lives of many older adults. The transmission there was solely human to human so the animal risk was limited. No outbreak in older adults could be traced back from agricultural animals or pet animals. In contrast, companion animals, farmed animals, and captive wild animals got infected with SARS-CoV-2 after having contact with asymptomatic or symptomatic humans. Nevertheless, animal-to-human transmission is likely only a minor route, if any, of transmission for SARS-CoV-2.

In view of the large number of COVID-19 victims among older adults, minimizing the SARS-CoV-2 risk for seniors has to be prioritized over protection of the general public. In the particular situation of older adults keeping companion dogs or cats, a general warning against the potential pet-to-human transmission is still warranted, despite the low risk based on experimental and epidemiological data. While the SARS-CoV-2 transmission risk is particularly low between dogs and humans, there may exist species-specific susceptibilities in cats, ferrets, or wild mustelids, which however we are only beginning to observe. In many countries, effective vaccination programs start to provide protection to the most vulnerable and exposed people in the population. Yet, the new virus variants emerging from South Africa, Brazil, and the UK are thought to possess a change in the virus’s spike protein that can allow the virus to even more easily enter cells. Thus, it is possible that SARS-CoV-2 will be prevalent within the human and the animal populations for the foreseeable future. Animals may theoretically play a role by either establishing a reservoir for new strains of SARS-CoV-2 and infected companion animals are also potentially able to spread new strains of SARS-CoV-2 to other people and pets in the household. Thus, people should be continuously advised to always follow standard handwashing practices before and after interacting with animals.

## Data Availability

Not applicable.
